# Impact of Na^+^ permeation on collective migration of pulmonary arterial endothelial cells

**DOI:** 10.1371/journal.pone.0250095

**Published:** 2021-04-23

**Authors:** Ningyong Xu, Linn Ayers, Viktoriya Pastukh, Mikhail Alexeyev, Troy Stevens, Dhananjay T. Tambe

**Affiliations:** 1 Department of Physiology and Cell Biology, College of Medicine, University of South Alabama, Mobile, Alabama, United States of America; 2 Center for Lung Biology, College of Medicine, University of South Alabama, Mobile, Alabama, United States of America; 3 Departments of Internal Medicine, College of Medicine, University of South Alabama, Mobile, Alabama, United States of America; 4 Departments of Pharmacology, College of Medicine, University of South Alabama, Mobile, Alabama, United States of America; 5 Department of Mechanical, Aerospace, and Biomedical Engineering, College of Engineering, University of South Alabama, Mobile, Alabama, United States of America; Indiana University School of Medicine, UNITED STATES

## Abstract

Collective migration of endothelial cells is important for wound healing and angiogenesis. During such migration, each constituent endothelial cell coordinates its magnitude and direction of migration with its neighbors while retaining intercellular adhesion. Ensuring coordination and cohesion involves a variety of intra- and inter-cellular signaling processes. However, the role of permeation of extracellular Na^+^ in collective cell migration remains unclear. Here, we examined the effect of Na^+^ permeation in collective migration of pulmonary artery endothelial cell (PAEC) monolayers triggered by either a scratch injury or a barrier removal over 24 hours. In the scratch assay, PAEC monolayers migrated in two approximately linear phases. In the first phase, wound closure started with fast speed which then rapidly reduced within 5 hours after scratching. In the second phase, wound closure maintained at slow and stable speed from 6 to 24 hours. In the absence of extracellular Na^+^, the wound closure distance was reduced by >50%. Fewer cells at the leading edge protruded prominent lamellipodia. Beside transient gaps, some sustained interendothelial gaps also formed and progressively increased in size over time, and some fused with adjacent gaps. In the absence of both Na^+^ and scratch injury, PAEC monolayer migrated even more slowly, and interendothelial gaps obviously increased in size towards the end. Pharmacological inhibition of the epithelial Na^+^ channel (ENaC) using amiloride reduced wound closure distance by 30%. Inhibition of both the ENaC and the Na^+^/Ca^2+^ exchanger (NCX) using benzamil further reduced wound closure distance in the second phase and caused accumulation of floating particles in the media. Surprisingly, pharmacological inhibition of the Ca^2+^ release-activated Ca^2+^ (CRAC) channel protein 1 (Orai1) using GSK-7975A, the transient receptor potential channel protein 1 and 4 (TRPC1/4) using Pico145, or both Orai1 and TRPC1/4 using combined GSK-7975A and Pico145 treatment did not affect wound closure distance dramatically. Nevertheless, the combined treatment appeared to cause accumulation of floating particles. Note that GSK-7975A also inhibits small inward Ca^2+^ currents via Orai2 and Orai3 channels, whereas Pico145 also blocks TRPC4, TRPC5, and TRPC1/5 channels. By contrast, gene silence of Orai1 by shRNAs led to a 25% reduction of wound closure in the first 6 hours but had no effect afterwards. However, in the absence of extracellular Na^+^ or cellular injury, Orai1 did not affect PAEC collective migration. Overall, the data reveal that Na^+^ permeation into cells contributes to PAEC monolayer collective migration by increasing lamellipodial formation, reducing accumulation of floating particles, and improving intercellular adhesion.

## Introduction

Collective migration of endothelial cells is an essential process during angiogenesis and wound healing [1]. In an *in vitro* wound closure assay, cells at the wound edge lead the closure by protruding lamellipodia into the wounded area. Migrating cells constantly reorganize their cytoskeleton while maintaining intercellular adhesion and monolayer integrity [2–8].

Lamellipodial dynamics, cytoskeletal activity, and intercellular adhesion are regulated through Na^+^ and Ca^2+^ signals [7, 9–14]. Na^+^ permeation channels implicated in endothelial migration include stretch-sensitive ENaC, NCX, Na^+^/H^+^ exchanger 1 (NHX1), Orai1, and TRPC1/4 [15–21]. Several of these channels regulate lamellipodial protrusion. For example, scratch-induced activation of ENaC in wound-edge cells is a key trigger for lamellipodial formation [15, 17, 22]. Na^+^ influx through ion channels/transporters located at the front of migrating cells induces water following via aquaporin that facilitates membrane expansion and the formation of lamellipodia [11, 18, 23]. Na^+^ influx through ENaC is necessary to steer cell migration in galvanotaxis [24]. The effect of Na^+^ permeation on cell migration is co-dependent on Ca^2+^ ions in two primary ways. The first way involves the regulation of both Na^+^ and Ca^2+^ movements. For example, Orai1 constitutively inhibits both the baseline Na^+^ leak and agonist-induced Na^+^ entry, but this effect involves Ca^2+^ displacing Na^+^ from entering the channel pore [25, 26]. Another example includes NCX which uptakes Na^+^ and extrudes Ca^2+^ in its forward mode [27, 28]. During wound healing, NCX functionally couples to ENaC and operates in reverse mode, partially contributing to slow the Ca^2+^ wave [15]. The second way of co-dependence involves the need for Ca^2+^ to sustain cellular migration. For example, Ca^2+^ ions are crucial in order to sustain migration-associated reorganization of actin cytoskeleton and contractile actomyosin activity [7, 14, 29]. Ca^2+^ ions regulate contractile forces that steer collective cell migration, cytoskeletal remodeling that sustain lamellipodial activity, and intercellular adhesion that maintains barrier integrity [10, 14, 25, 26, 30–32]. Altering cellular Na^+^ and Ca^2+^ slows endothelial wound closure [7, 9, 23]https://www.zotero.org/google-docs/?zFxE5M.

In a variety of endothelial cell lines, previous studies have focused on the role of Na^+^ permeation in the migration-associated cellular events. However, in PAEC wound closure, the effect of blocking Na^+^ entry or disrupting selective permeation associated with ENaC, NCX, Orai1, and TRPC1/4 remains unexplored. To fill this knowledge gap, here we used scratch wound closure and unscratched gap closure assays and examined the effect of Na^+^ transport on collective migration of PAEC over 24 hours.

## Materials and methods

### Cell preparation

#### Isolation and culture of rat pulmonary artery endothelial cells

PAECs were isolated from rat lungs and cultured as described by Stevens and colleagues [31]. Briefly, wild-type rat PAECs were isolated and cultured in growth media which included regular Dulbecco’s Modified Eagle Media (DMEM, 156 mM Na^+^, catalog no. 11965, Invitrogen), 8% fetal bovine serum (FBS, catalog no. 10082147, Invitrogen) and 1% penicillin-streptomycin (catalog no. 15140–122, Invitrogen). Cells were incubated in a humidified 37°C incubator (Forma 3130 CO_2_ HEPA Incubator, Thermal Scientific) supplied with 5% CO_2_.

For migration studies, confluent PAECs were trypsinized using 0.05% trypsin (containing 0.02% EDTA, catalog no. 25300–054, Invitrogen), and about 1.5 × 10^5^ to 2.1 × 10^5^ cells in 2 mL growth media were plated in a 35 mm cell culture dish (catalog no. 430165, Corning). The cells were grown to confluence over 3–4 days in a humidified tissue culture environment (37°C, 5% CO_2_).

#### Gene silence of Orai1

Endothelial cells were engineered for conditional silence of Orai1 as described previously [25, 26, 33]. Briefly, recombinant retro- and lentiviruses were generated, and the PA2641 and PA2879 cell lines were established. Wild-type rat PAECs were transduced with rv.2641, a retrovirus strain encoding a Tet-On reverse tetracycline transactivator protein (rtTA), followed by an internal ribosomal entry site, an enhanced green fluorescent protein (EGFP), and a blasticidin resistance gene [33]. Transduced PA2641 cells were kept in Tet growth media [regular DMEM supplemented with 8% Tet system approved FBS (tFBS, catalog no. 631101, Clontech) and 1% penicillin-streptomycin] and selected to homogeneity by adding 10 μg/mL blasticidin (catalog no. ant-bl-1, InvivoGen). The purity of the PA2641 cells transduced with rv.2641 was confirmed by observing homogenous EGFP fluorescence. Purified cells were then transduced with a lentivirus strain lv.2879 which encodes mCherry and a doxycycline-inducible Orai1-targeted shRNA [26]. Double transduced PA2879 cells were purified to homogeneity by adding 1 μg/mL puromycin (catalog no. 9620, Sigma). Cellular purity was confirmed by homogeneously expressing mCherry reporter after being treated with 3 μg/mL doxycycline (catalog no. 631311, Clontech).

A confluent monolayer of purified PA2879 cells was trypsinized from a T75 flask (catalog no. 10-126-37, Fisher Scientific) and plated into 35 mm dishes with seeding densities between 2 × 10^5^ and 3 × 10^5^ cells / dish. Each dish of cells was treated with 2 mL Tet growth media and stored in a humidified 37°C incubator supplied with 5% CO_2_ for one day. In the following day, cells were daily treated with freshly prepared doxycycline 3 μg/mL in Tet growth media for 72 hours while cells were growing to confluence. Doxycycline-untreated cells were used as control. The silence of Orai1 was confirmed by western blot assay using whole cell lysates in a doxycycline concentration- and time-dependent manner [25]. Red fluorescence images were also taken to confirm the expression of mCherry under a doxycycline-treated condition, supporting the evidence of Orai1 silencing [26].

### Assessment of collective migration

#### Customized choline-DMEM

To assess the overall effect of extracellular Na^+^ on collective migration, we plan to use Na^+^-depleted media during migration. Based on the regular DMEM which contained 156 mM Na^+^, all Na^+^-containing ingredients were substituted with choline-containing ingredients by Invitrogen Company following our request. The modified ingredients of choline-DMEM were (mM): choline chloride 111.27, KCl 4.43, choline bicarbonate 44.05, and KH_2_PO_4_ 123.27. The rest of the components, including CaCl_2_ 1.8 mM, the osmolarity (320–340 mOsm), and the pH (6.95–7.2), were maintained the same as in the regular DMEM.

#### Migration media

Migration media were composed of the Na^+^- or choline-containing DMEM supplemented with 1% penicillin-streptomycin and 2% FBS. For wild-type PAECs, we added regular cell culture FBS (catalog no. 10082147, Invitrogen). For genetically modified PA2879 cells, we added tFBS. For pharmacological inhibition approaches, we added FBS (catalog no. S11550H, Atlanta).

#### Migration assays

To assess the effect of scratch-associated cellular injury on collective migration, we used two different migration assays as described below. In wound closure assay, we created a wound for cells to migrate by scratching the center of a PAEC confluent monolayer. In gap closure assay, we created a gap for cells to migrate by removing a barrier between two adjacent monolayers.

**Fig 1:**
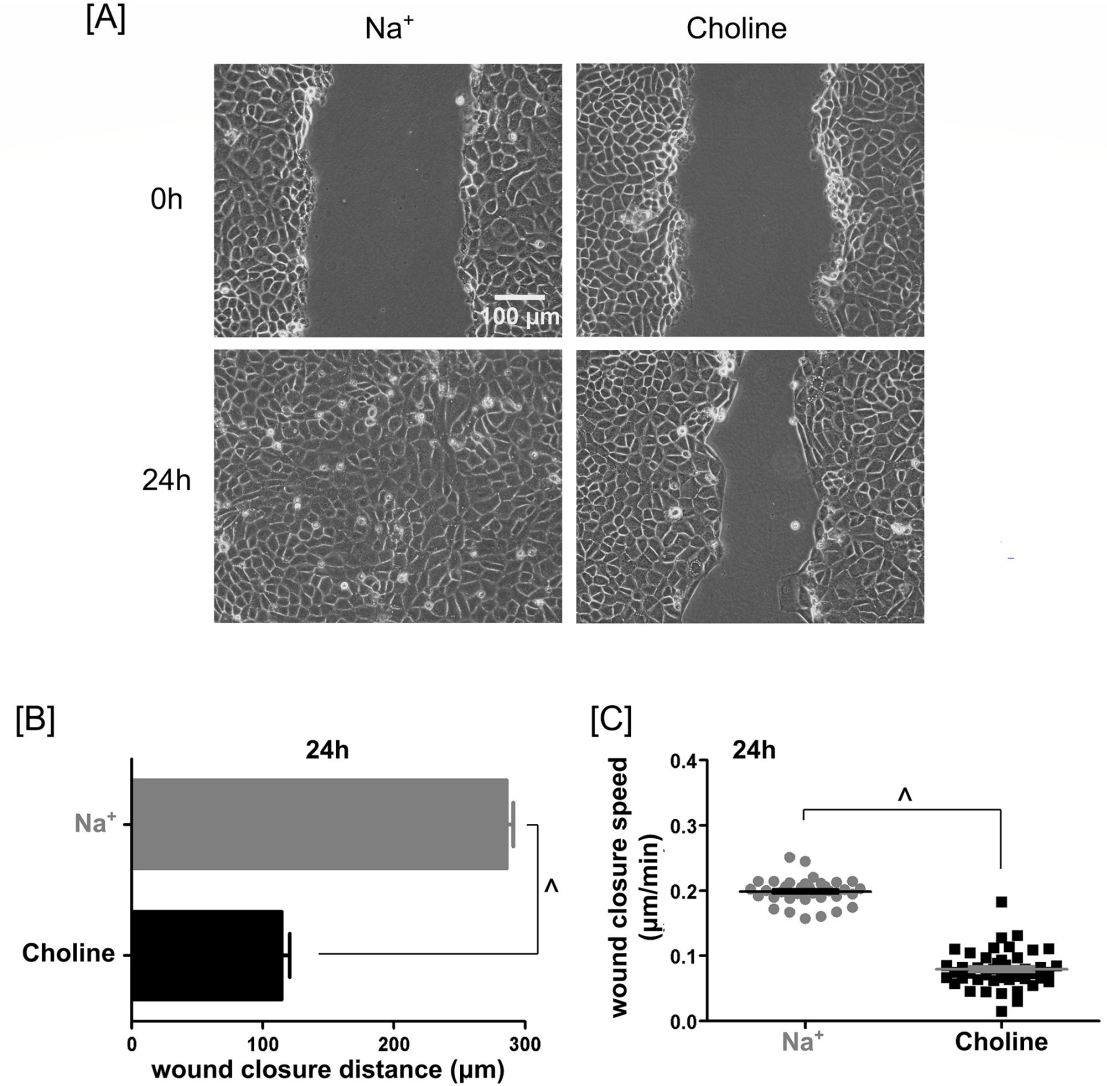
Contribution of extracellular Na^+^ to PAECs scratch wound closure. **(A)** Phase-contrast images of PAEC monolayers. The top and bottom rows showed monolayers immediately after and 24 hours after scratching, respectively. The monolayers in the left and right columns were in a Na^+^- and choline-containing media, respectively. In the absence of extracellular Na^+^, wound closure was incomplete at 24 hours. **(B)** Bar plot of average wound closure distances over 24 hours in Na^+^- and choline-containing media. Over 24 hours, extracellular Na^+^ enabled PAECs to migrate farther. **(C)** Scatter plot of wound closure speeds in both Na^+^- and choline-containing media. The migration speed of PAECs was significantly slower in the absence of extracellular Na^+^ when compared to the Na^+^-containing counterpart. Both the bar plot and scatter plot showed mean ± SEM. Statistical significance was assessed using unpaired *t*-test with Welch’s correction (^—*P* < 0.0001).

*Wound closure assay*. To examine the effect of extracellular Na^+^ on wound closure, PAEC monolayer was scratched at the center using a sterile pipette tip (0.5–10 μL tip, catalog no. 4830, Coring) (Fig 1A). Before starting the image acquisition, the monolayer was immediately rinsed three times with Na^+^- or choline-containing DMEM and kept in 2 mL of the corresponding migration media.

To examine the effect of inhibiting specific Na^+^ and Ca^2+^ channels on wound closure, PAECs were seeded at 5 × 10^5^–6 × 10^5^ cells / well in 6-well dishes (catalog no. 3506, Corning) and grown to confluent monolayers in growth media containing 8.6% FBS (catalog no. S11550H, Atlanta). A scratch was generated at the center of each well using a sterile p200 pipette tip. (catalog no. 11110706, USA Scientific Inc). Before starting the image acquisition, monolayers were gently rinsed three times with treatments and kept in 2 mL of the same treatments. The treatments contained Na^+^-containing migration media and one or two channel inhibitor(s) [10 μM benzamil (catalog no. B2417, Sigma), 90 μM amiloride (catalog no. 129876, Sigma), 10 μM GSK-7975A (catalog no. AOB4124, AOBIOUS), and/or 1 nM Pico145 (catalog no. HY-101507, MedChemExpress)] [34–38]. Dimethyl Sulfoxide (DMSO, 1:1371 dilution, catalog no. D2438, Sigma) was used as vehicle control.

To assess the effect of Orai1 expression on wound closure, Orai1-expressing and -silenced PA2879 monolayers were scratched at the center with sterile pipette tips (0.5–10 μL tip, catalog no. 4830, Corning). Before starting the image acquisition, the monolayer was rinsed three times with Na^+^- or choline-containing DMEM and kept in 2 mL of the corresponding migration media.

*Gap closure assay*. A sterile silica culture-insert (catalog no. 80209, ibidi) was firmly pushed down onto a sterile 35 mm cell culture dish to form two sealed wells separated by a 500-μm-thick wall. The size of each well was approximately 8 mm × 3.5 mm. In each well, between 2.8 × 10^4^ and 3.5 × 10^4^ PA2879 cells were seeded with 70 μL Tet growth media. From the next day, cells in the wells were daily treated with freshly prepared doxycycline 3 μg/mL in Tet growth media for 72 hours while cells were growing to confluence. Doxycycline-untreated cells were used as control. Thereafter, the culture-insert was lifted from the dish to create a gap between two small patches of confluent cells. Before starting the image acquisition, the patches of cells were gently rinsed three times with Na^+^- or choline-containing DMEM and kept in 2 mL of the corresponding migration media.

It is important to note that in wound closure assay a single scratch was made at the center of a confluent monolayer that covered the entire 35 mm dish. However, in gap closure assay, a gap was formed between two small patches of confluent cells.

### Assessment of cytosolic Ca^2+^ and Na^+^ after scratching

To investigate the contribution of Orai1 activity to scratch-induced changes in cytosolic Ca^2+^ and Na^+^, PAECs were grown on a 25-mm diameter coverglass (catalog no. 12-545-102, Fisherbrand) that was suspended in a 35 mm dish. Once the cells grew to confluence after 4–5 days, the monolayer was rinsed three times with Krebs-Henseleit (Krebs) buffer (catalog no. K3753, Sigma, ~156 mM Na^+^, 290 mOsm, pH 7.4) containing 2 mM CaCl_2_ (catalog no. 223506, Sigma) and 25 mM HEPES (catalog no. 3375, Sigma). The monolayer was incubated at 37°C for 30 minutes with 6.315 μM Cal-590 AM (catalog no. 20510, AAT Bioquest) or 9.225 μM Ion NaTRIUM Green-2 AM (catalog no. ab142802, Abcam) in 1 mL Krebs buffer containing 8 μL Pluronic F-127 (catalog no. P6866, Life Technologies). The monolayer was then washed twice and incubated with 10 μM GSK-7975A or vehicle control (DMSO 1:1371 dilution) in 1 mL Krebs buffer at room temperature for 15 minutes. Finally, the coverglass was assembled into an Attofluor cell chamber (catalog no. A7816, Invitrogen), and 2 mL fresh 10 μM GSK-7975A or vehicle control was added. Scratching was done immediately before time-lapse imaging.

### Image acquisition protocols

#### Time-point image acquisition

To examine the effects of extracellular Na^+^ and/or Orai1 silencing on collective migration, the migrating monolayer was imaged at 0, 6 and 24-hour time points using an inverted epifluorescence microscope (Olympus IX70). In the interim, the cells were stored in a tissue culture incubator. The images were acquired using a 20× LCPlanFl objective and a SPOT camera controlled by SPOT version 4.0.9 software. The same setup was used to examine the effect of Orai1 expression on collective migration as well.

To examine the effect of inhibiting specific Na^+^ and Ca^2+^ entry channels on wound closure, the migrating monolayer was imaged at 0, 6 and 24-hour time points using an inverted epifluorescence microscope (Nikon Eclipse TE2000-S). In the interim, the cells were stored in a tissue culture incubator. The images were acquired using a 10× Nikon Ph1 ADL objective and a Hamamatsu camera (ORCA-Flash4.0 V3) controlled via μManager version 2.0 software.

#### Time-lapse image acquisition

To examine the effect of extracellular Na^+^ on wound closure, the migrating monolayer was imaged every 10 minutes for 24 hours using an inverted epifluorescence microscope (Nikon Eclipse TE2000-U). The microscope was equipped with a stage top incubator (LiveCell^™^ Stage Top Incubation System, Pathology Device, Inc., Westminster, MD, USA). The atmosphere inside the incubator was maintained at 37°C and supplied with humidified 5% CO_2_. The images were acquired using a 10× Nikon CFI Plan Fluor Ph1 DLL objective and a Photometrics CoolSNAP ES camera controlled by NIS Elements version 3.10 software.

To examine cytosolic Ca^2+^ and Na^+^ changes after scratching, five baseline fluorescence images were taken before scratching at random spots in a confluent monolayer. The monolayer was wounded by scraping off cells with a sterile p200 pipette tip (catalog no. 11110706, USA Scientific Inc). Washing was omitted. The monolayer area encompassing the wound edge was immediately imaged every 5 seconds for 30 minutes using an inverted epifluorescence microscope (Nikon Eclipse TE2000-S). Fluorescence images were acquired using a 20× Nikon Plan Fluo objective, a Lambda DG4 laser light source, and a Hamamatsu camera (ORCA-Flash4.0 V3) controlled via an acquisition module of iTACS software [39].

### Analysis

#### Image analysis

Scratch wound closure distance, speed, frequency distribution of speed, change in speed, and unscratched gap closure distance were measured using National Institutes of Health ImageJ software (http://rsb.info.nih.gov/ij/). Cytosolic Ca^2+^ and Na^+^ intensities were quantified using ImageJ and an analysis and visualization module of iTACS [39, 40].

**Fig 2:**
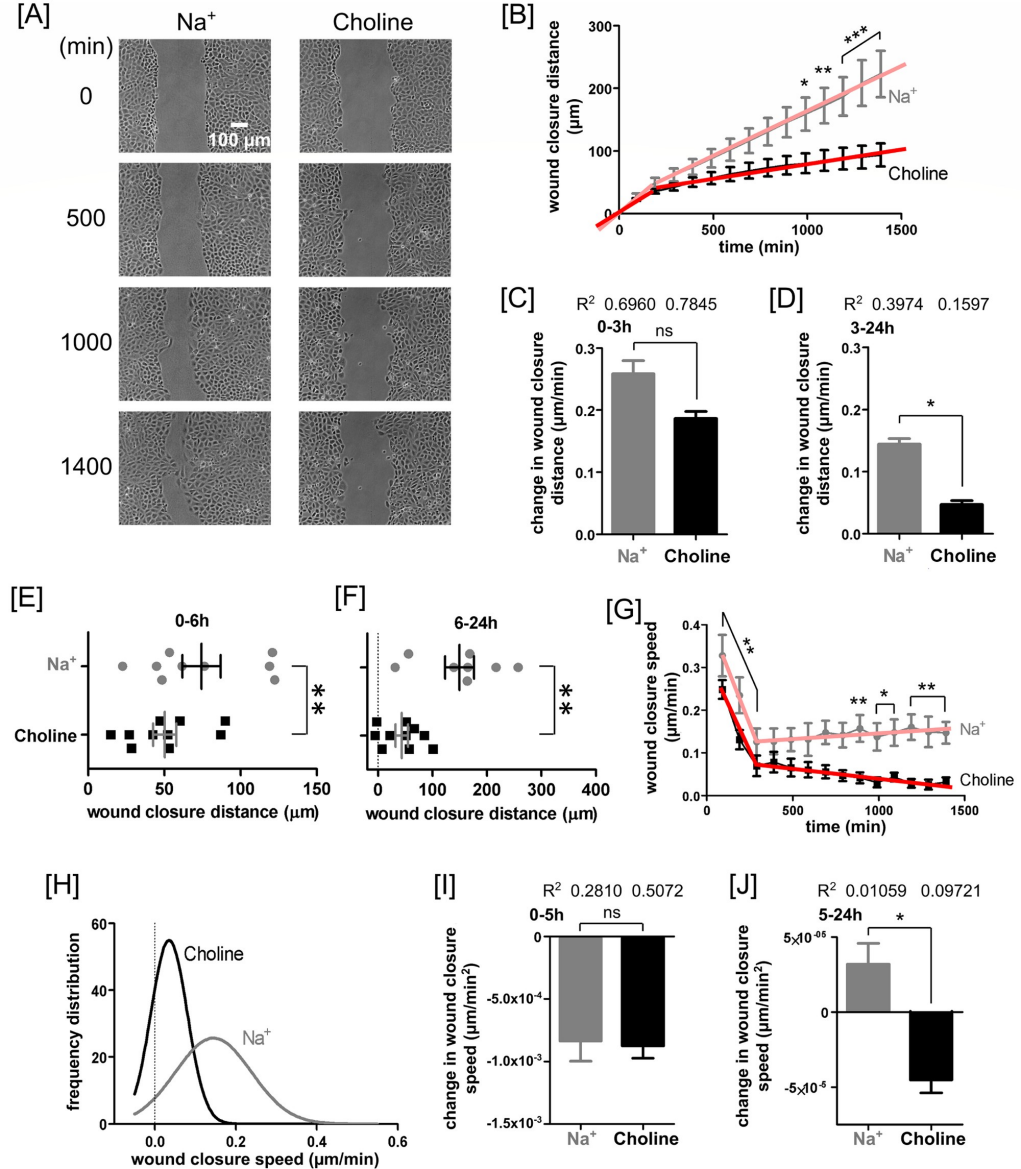
Contribution of extracellular Na^+^ to the 24-hour progression of PAEC wound closure. **(A)** Sequential phase-contrast images of PAEC monolayers show wound closure in Na^+^- and choline-containing media. The wound closure was faster in Na^+^ than in choline condition. **(B)** The plot of wound closure distance as a function of time in a Na^+^- and choline-containing media. At each time point, wound closure distances were always quantified from time 0. In both conditions, wound closure occurred in two phases. These phases were analyzed using two linear regression fits, one from 0 to 190 minutes and other from 190 to 1390 minutes. The wound closure distance between 990 and 1390 minutes was significantly larger in Na^+^ condition than in choline condition. Bar plot of the slope of **(C)** the first linear regression fit (time 0 to 190 minutes), and **(D)** the second linear regression fit (time 190 and 1390 minutes) through the wound closure distances in Na^+^- and choline-containing media. During both phases, the wound closure distance in choline-containing media trailed that in the Na^+^-containing media. However, the difference was statistically significant only for the second phase. Scatter plots of wound closure distance in (**E**) 0–6 hours (or more accurate 0–390 minutes) and (**F**) 6–24 hours (**—*p* < 0.01, Wilcoxon matched paired *t*-test). **(G)** A plot of wound closure speed as a function of time quantified at a 100-minute interval. This plot also exhibited two linear phases. In the first phase, which spans 0–290 minutes, the wound closure speed decreased rapidly. In the second phase, which spans 290–1390 minutes, the wound closure speed changed minimally. Across the entire timespan, extracellular Na^+^ appeared to facilitate faster cell migration. **(H)** Distribution of wound closure speed displayed using Gaussian fit through the data. The distributions showed that extracellular Na^+^ increased variability in wound closure speed. Bar plot of the slope of **(I)** the first linear regression fit (time 0 to 290 minutes), and **(J)** the second linear regression fit (time 290 and 1390 minutes) through the wound closure speeds in Na^+^- and choline-containing media. During the first phase, the change in wound closure speed was similar in both conditions. However, during the second phase, the wound closure speed decreased in choline-containing media and increased in Na^+^-containing media. The data include at least 8 repeat experiments contributing to at least 8 images for each time point. Panels **(B)**-**(G)**, **(I)**, and **(J)** show mean values with standard errors. Statistical significance was assessed using two-way ANOVA followed by *Bonferroni post hoc* test and linear regression (ns—not significant, *—*P* < 0.05, **—*P* < 0.01, ***—*P* < 0.001, ^—*P* < 0.0001).

*Quantitative assessment of wound closure distance and speed*. From the sequence of images of a migrating monolayer, the wound width and the speed of wound closure was quantified for one out of every 10 images (100 minutes) (Fig 2). Pixel coordinates defining the boundary of a wound or gap area (pixel^2^) were identified using ImageJ. The wound width was calculated as follows:

$$Wound\;width(\mu m)=\frac{Wound\;or\;gap\;area(pixel^{2})}{Image\;height\left(pixel\right)}\times Pixel\;size(\mu m/pixel)$$ (1)

Wound closure distance was computed as a difference in the wound width at time 0 minutes (t = 0, i.e., when the imaging of the migration front started) and the wound width at any subsequent time (t>0).

$$Wound\;closure\;distance(\mu m)=Wound\;width_{t=0}(\mu m)–Wound\;width_{t>0}(\mu m)$$ (2)

The wound or gap closure speed (μm/min) was defined as a ratio of wound closure distance and time taken to travel that distance.

$$\begin{array}{l} Wound\;closure\;speed_{t}\\ \left(\mu m/min\right) \end{array}=\frac{\left(Wound\;closure\;distance_{t+50\;min}–Wound\;closure\;distance_{t-50\;min})(\mu m\right)}{100\left(min\right)}$$ (3)

*Quantification of cytosolic Ca*^*2+*^ *and Na*^*+*^ *intensity and cell movements*. Average cytosolic Ca^2+^ and Na^+^ intensity, and median speed of cell movements were quantified within a narrow band located progressively farther from the migration front (S2 Fig [https://doi.org/10.6084/m9.figshare.13818989]). Normalized fluorescence intensity of Ca^2+^ and Na^+^ were quantified as follows:

$${\left[Ca^{2+}\right]}_{i}\;or\;{\left[Na^{+}\right]}_{i}=\frac{F_{0}–F_{30}}{F_{B}{}_{L}}$$ (4)

where *F*_0_ is fluorescence intensity immediately after the scratch, *F*_30_ is fluorescence intensity 30 min after the scratch, and *F*_*BL*_ is baseline fluorescence intensity before the scratch.

#### Statistical analysis

*Sample size*. To assess the effect of extracellular Na^+^ on endothelial scratch wound closure, we conducted 4 repeats of paired experiments with wild-type PAECs both in the presence and absence of extracellular Na^+^. Collectively these experiments contributed 35 images in Na^+^- and 45 images in choline-containing media.

To assess the dynamics of endothelial scratch wound closure, we conducted 8–10 repeats of paired time-lapse experiments both in the presence and absence of extracellular Na^+^ for 24 hours. We quantified a total of 120 images in Na^+^ and 150 images in choline condition.

To assess the effect of pharmacological inhibition of specific Na^+^ and Ca^2+^ channels on endothelial scratch wound closure, we conducted 3–6 repeats of paired experiments at 0, 6, and 24-hour time points, using channel inhibitors or vehicle control. From these experiments, we analyzed 30–60 images at each time point in each condition.

To assess the effect of Orai1 silencing on endothelial scratch wound closure, we conducted 8 repeats of paired experiments at 0-hour time point and 7 repeats at both 6- and 24-hour time points in each of the four conditions: Orai1-expressing and -silenced PA2879 cells in the presence and absence of extracellular Na^+^. We analyzed 60–78 images at each time point in each condition.

To assess the effect of Orai1 silencing on endothelial unscratched gap closure (i.e., migration initiated without scratch injury), we conducted 7–9 repeats of paired experiments at 0, 6, and 24-hour time points in each of the four conditions: Orai1-expressing and -silenced PA2879 cells in the presence and absence of extracellular Na^+^. We quantified 30–47 images at each time point in each condition. More importantly, we compared the results with the wound closure results to determine the contribution of scratch to collective migration.

To assess the scratch-triggered changes in cytosolic Ca^2+^, we conducted 2 repeats of paired experiments. To assess the changes in cytosolic Na^+^, we conducted one paired experiments. We analyzed 361 images in each experiment.

*Significance tests*. To compare the data across different time-points, we used an unpaired two-tailed Student’s *t*-test, if unspecified. To examine the statistical significance of the slope of the wound width or wound closure speed in PAECs, we used a two-way ANOVA with *Bonferroni post hoc* test and linear regression. Wilcoxon matched paired *t*-test was used to detect the significance of wound closure distance between Na^+^ and choline groups using data sets at 390 minutes and 24 hours in time-lapse studies. A one-way ANOVA with *Bonferroni post hoc* test was used to detect the significant difference in wound closure distances among vehicle control and several channel inhibitors. A one-way ANOVA with *Tukey’s post hoc* test was used to detect the significance of Orai1 and extracellular Na^+^ in scratch wound closure and unscratched gap closure. Data were expressed as mean values with standard errors of mean (mean ± SEM). To detect the significance of changes in Ca^2+^ and Na^+^ fluorescence in response to scratch in the presence and absence of GSK-7975A, we used the linear regression and one-way ANOVA with *Bonferroni post hoc* test. A value of *P* < 0.05 was considered statistically significant for all the comparisons. Datasets were plotted using GraphPad Prism 5.01 (GraphPad Software, Inc., La Jolla, CA, USA).

## Results

### Absence of extracellular Na^+^ slows PAEC monolayer scratch-wound closure

In 24 hours, PAECs in Na^+^-containing media healed 99% of the wound area (Fig 1). By contrast, PAECs in choline-substituted media healed only 37% of the wound area (Fig 1).

### PAEC scratch wounds close in two phases

Although PAEC scratch wounds closed much slower in the absence of extracellular Na^+^, there were two approximately linear phases of the wound closure processes both in the presence and absence of extracellular Na^+^ (Fig 2A and 2B). After quantifying the migration speed over 100 minutes intervals, we found actually the first linear phase was roughly from 0 to 5 hours, and the second linear phase was from 6 to 24 hours (Fig 2G). Over 24 hours, extracellular Na^+^-dependent cellular processes accounted for about 60% of the wound closure distance (Fig 2B). The average distance of wound closure per minute over the first 3 hours were not significantly different between Na^+^ and choline conditions (Fig 2C). By contrast, the difference was significant after 3 hours; PAEC monolayers closed significantly less wound distance in choline condition (Fig 2D). Time-point plots also revealed that Na^+^ depletion significant reduced wound closure distances by 33% and 71%, respectively, in the first 6 hours and between 6 and 24 hours (Fig 2E and 2F).

Besides the wound closure distance, the wound closure speed assessed using Eq 3 also revealed a biphasic progression (Fig 2G). By choosing a 100-minutes time interval in Eq 3, we focused on the phases rather than on the precise location where the first phase transitions into the second phase. The slope of the first phase, i.e., rate of change of wound closure distance, was steeper compared to that of the second phase. The influence of extracellular Na^+^ on the slope of the first phase was statistically insignificant, suggesting that the presence of extracellular Na^+^ did not prevent the reduction of wound closure speed. However, during this phase, PAEC wounds still closed significantly faster in Na^+^ than in choline condition. By contrast, during the second phase, the presence of extracellular Na^+^ sustained wound closure speed at a stable level, whereas lack of extracellular Na^+^ caused gradual speed decline to a significant extent.

In the presence of extracellular Na^+^, the wound closure speed exhibited greater heterogeneity (Fig 2H). The slope of the first phase, i.e., rate of change of wound closure speed, was steeper compared to that of the second phase. However, extracellular Na^+^ had no effect on the slope of the first phase (Fig 2I). By contrast, the slope of the second phase was positive in the presence—and negative in the absence—of extracellular Na^+^ (Fig 2J). As such, the wound closure was progressively faster in the presence—but progressively slower in the absence—of extracellular Na^+^. Such reduction in closure speed may be due to the absence of Na^+^-mediated wound-repair mechanisms.

### Absence of extracellular Na^+^ suppresses lamellipodia formation at the wound edge

**Fig 3:**
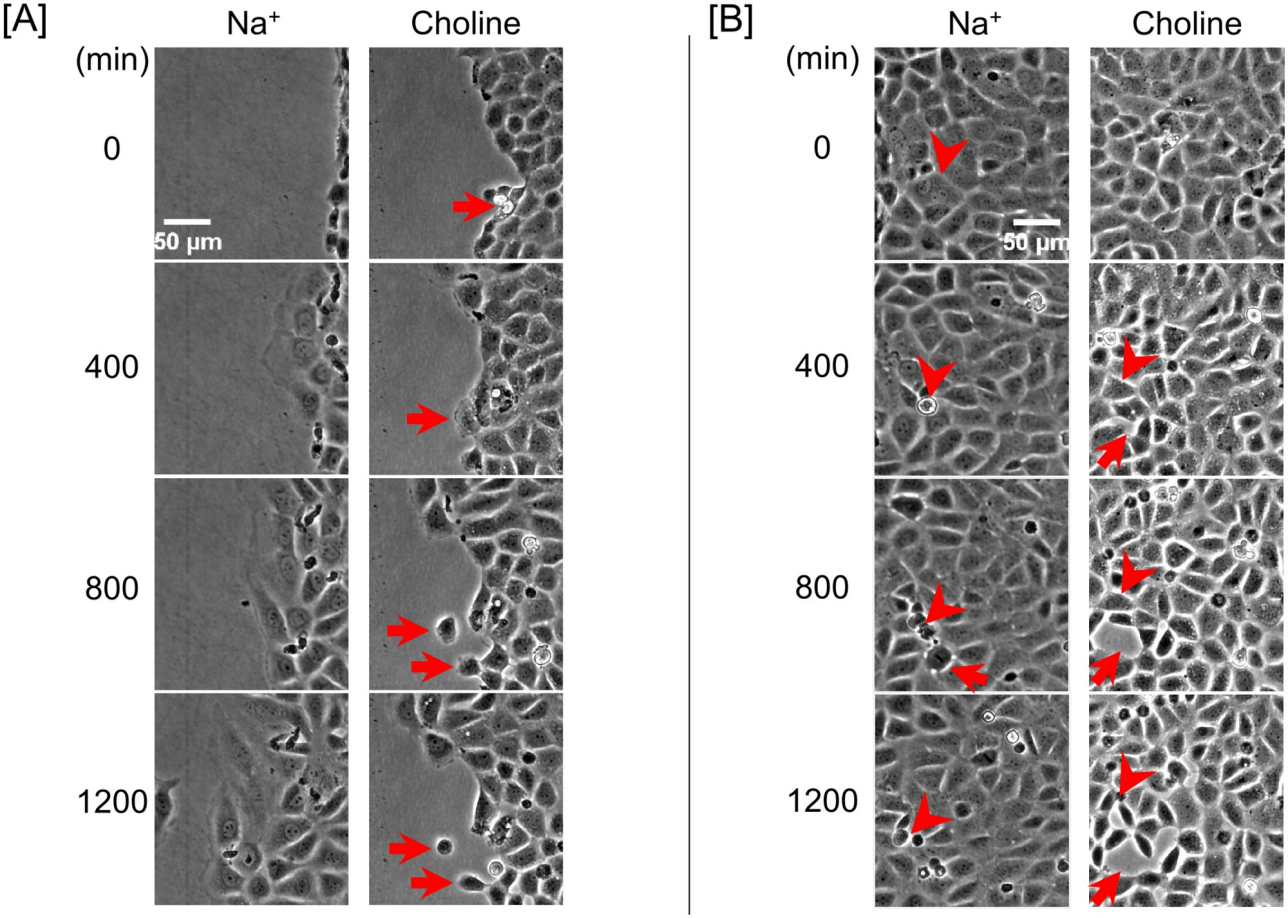
Contribution of extracellular Na^+^ to PAEC protruding lamellipodia, sustaining and reinstating intercellular adhesion, and advancing persistently. **(A)** Sequential phase-contrast images of a PAEC wound edge at 400-minute intervals in the presence and absence of extracellular Na^+^. PAECs at the leading edge protruded prominent and stable lamellipodia in Na^+^-containing media. In choline-containing media, fewer PAECs at the wound edge protruded lamellipodia, and most protruded lamellipodia were relatively smaller. Cells divided at the wound edge gradually formed interendothelial gaps and then detached from the monolayer. The detached individual cells thus lost lamellipodia and cell polarity, and eventually stopped migrating. **(B)** Sequential phase-contrast images of a confluent PAEC monolayer at 400-minute intervals in the presence and absence of extracellular Na^+^. In both conditions, transient gaps were both observed during cell divisions (arrowheads). Gaps were generally small and sealed quickly in Na^+^ condition (arrows). However, in choline condition, some sustained gaps were also observed. Some gaps formed even without cell division. The sustained gaps fused with adjacent newly formed gaps leading to gap size increase. Cell division instances are identified with arrowheads. Interendothelial gaps are identified with arrows.

Lamellipodia formation is necessary for cell migration. At the leading edge, some PAECs protruded prominent and stable lamellipodia in the presence of extracellular Na^+^ and migrated faster than others with smaller or no lamellipodia (Fig 3A and S1 Movie [https://doi.org/10.6084/m9.figshare.13818989]). Cells that formed prominent lamellipodia tended to migrate faster toward each other, no matter whether these cells were divided by the wound or in an adjacent area, suggesting that the existence of certain ion/chemotactic gradients which may be sensed by lamellipodia to stimulate crawling.

However, in choline-containing media, relatively fewer PAECs protruded lamellipodia at the wound edge, and the protruded lamellipodia were less prominent (Fig 3A and S2 Movie [https://doi.org/10.6084/m9.figshare.13818989]).

### Extracellular Na^+^ is necessary for PAECs to sustain and reinstate intercellular adhesion and to migrate persistently

In the presence of Na^+^, divided cells at the wound edge formed intact leading edge with the neighbor cells and steered monolayer continuously migrated into a wounded area (Fig 3A). In choline condition, some divided cells also formed an intact leading edge while others detached from the leading edge and became single cells. Some undivided wound-edge cells also detached from the monolayer while individually migrating forward, and they became single cells as well. These single cells lost lamellipodia and cellular polarity and thus stopped migrating.

In confluent PAEC monolayers, transient interendothelial gaps appeared around dividing cells immediately prior to cell division in both Na^+^- and choline-containing media (Fig 3B, and S1 and S2 Movies [https://doi.org/10.6084/m9.figshare.13818989]). In choline condition, interendothelial gaps also formed when there was no cell division. Gaps that formed in Na^+^ condition were very transient and generally very small; they resealed promptly. However, in choline condition, interendothelial gap resealing was remarkably slowed. Some sustained interendothelial gaps progressively increased in size over time and fused with adjacent gaps.

Altogether, these findings suggest that PAECs need Na^+^-dependent signaling mechanisms to protrude lamellipodia, quickly reseal interendothelial gaps, maintain intercellular adhesion, and migrate persistently. Thus, extracellular Na^+^ improves PAEC monolayer barrier integrity.

### Inhibition of Na^+^ permeation through ENaC slows wound closure

**Fig 4:**
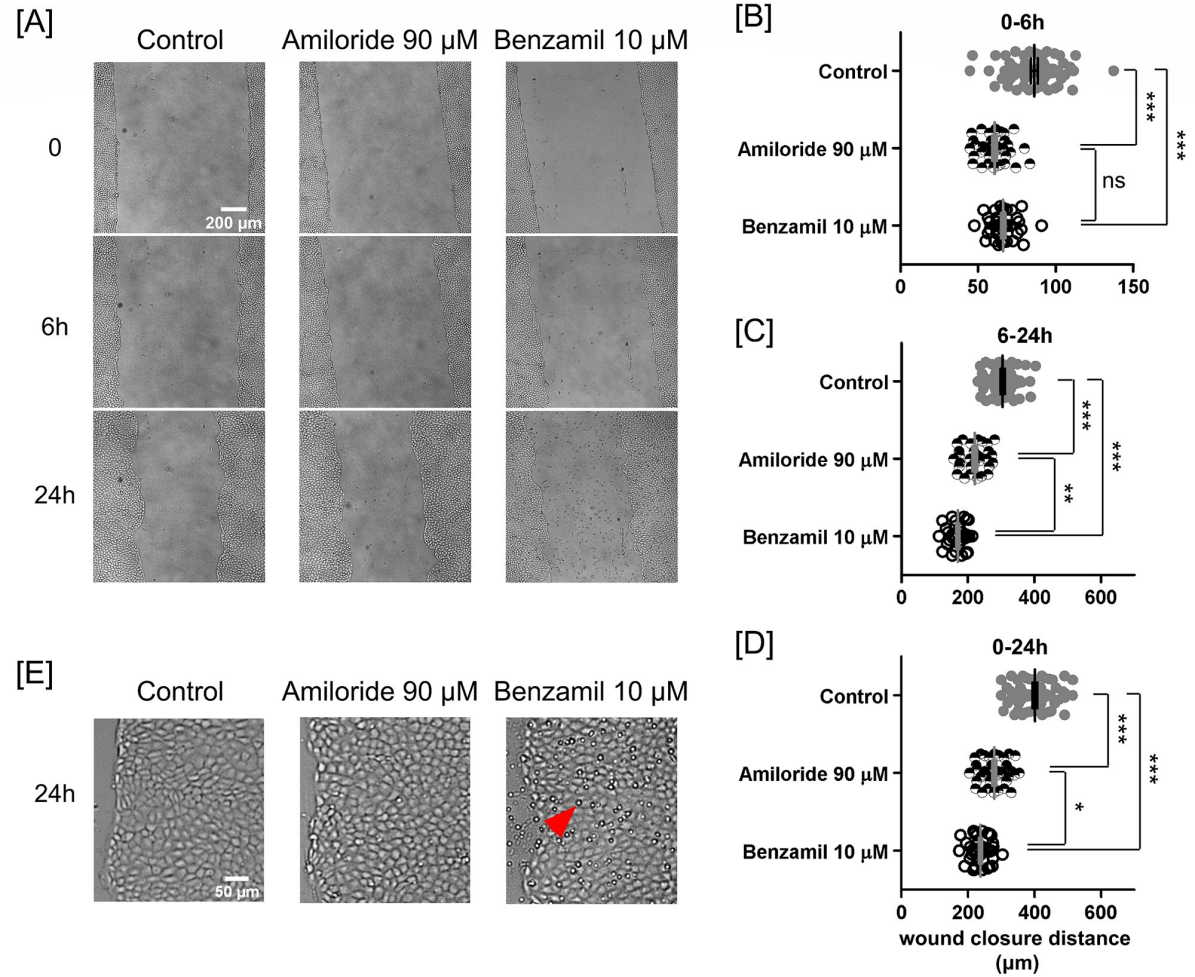
Inhibition of Na^+^ permeation through ENaC and NCX channels slowed PAEC scratch wound closure. **(A)** Brightfield images of scratched PAEC monolayers at 0, 6, and 24-hours in vehicle control, 90 μM amiloride, and 10 μM benzamil conditions. Benzamil treatment caused accumulation of floating particles over 24 hours. Studies were all conducted in Na^+^-containing media. Scatter plots of wound closure distance in **(B)** 0–6, **(C)** 6–24, and **(D)** 0–24 hours. Benzamil was more potent than amiloride to slow PAEC monolayer wound closure. Data were reported as mean ± SEM. **(E)** Magnified view of the marginal regions of the control, amiloride-treated, and benzamil-treated cells after 24 hours of migration. An arrowhead points to a floating particle. Statistical significance was assessed using one-way ANOVA with *Bonferroni post hoc* test (ns—not significant, *—*P* < 0.05, **—*P* < 0.01, ***—*P* < 0.0001).

To assess the effect of Na^+^ permeation through the ENaC on PAEC monolayer wound closure, we inhibited ENaC with 90 μM amiloride in the presence of extracellular Na^+^ [36, 37]. Such treatment significantly slowed PAEC monolayer scratch wound closure by 30% and 28%, respectively, in the first 6 hours and between 6 and 24 hours (Fig 4A–4C).

### Inhibition of Na^+^ permeation through NCX appears to delay the second phase of wound closure

To assess the effect of Na^+^ permeation through both ENaC and NCX on PAEC monolayer wound closure, we inhibited these two channels with 10 μM benzamil in the presence of extracellular Na^+^ [37, 38]. Although benzamil slowed 23% of wound closure when compared to control in the first 6 hours, additional inhibition of NCX cause no significant difference when compared to amiloride treatment (Fig 4B). Between 6 and 24 hours, benzamil slowed wound closure by 44% and 23%, respectively, when compared to control and amiloride treatment (Fig 4C). Overall, the benzamil-treated PAEC monolayers closed the least wound distance (Fig 4D).

### Inhibition of Na^+^ permeation through both ENaC and NCX channels causes accumulation of floating particles in media

Over 24 hours, only the benzamil-treated monolayers, rather than the control and amiloride-treated monolayers, released floating particles into the media (Fig 4A and 4E). These observations suggest that inhibiting both ENaC and NCX might severely weaken cellular ability to adhere, and perhaps, survive.

### Inhibition of Orai1 and TRPC1/4 does not affect overall wound closure

**Fig 5:**
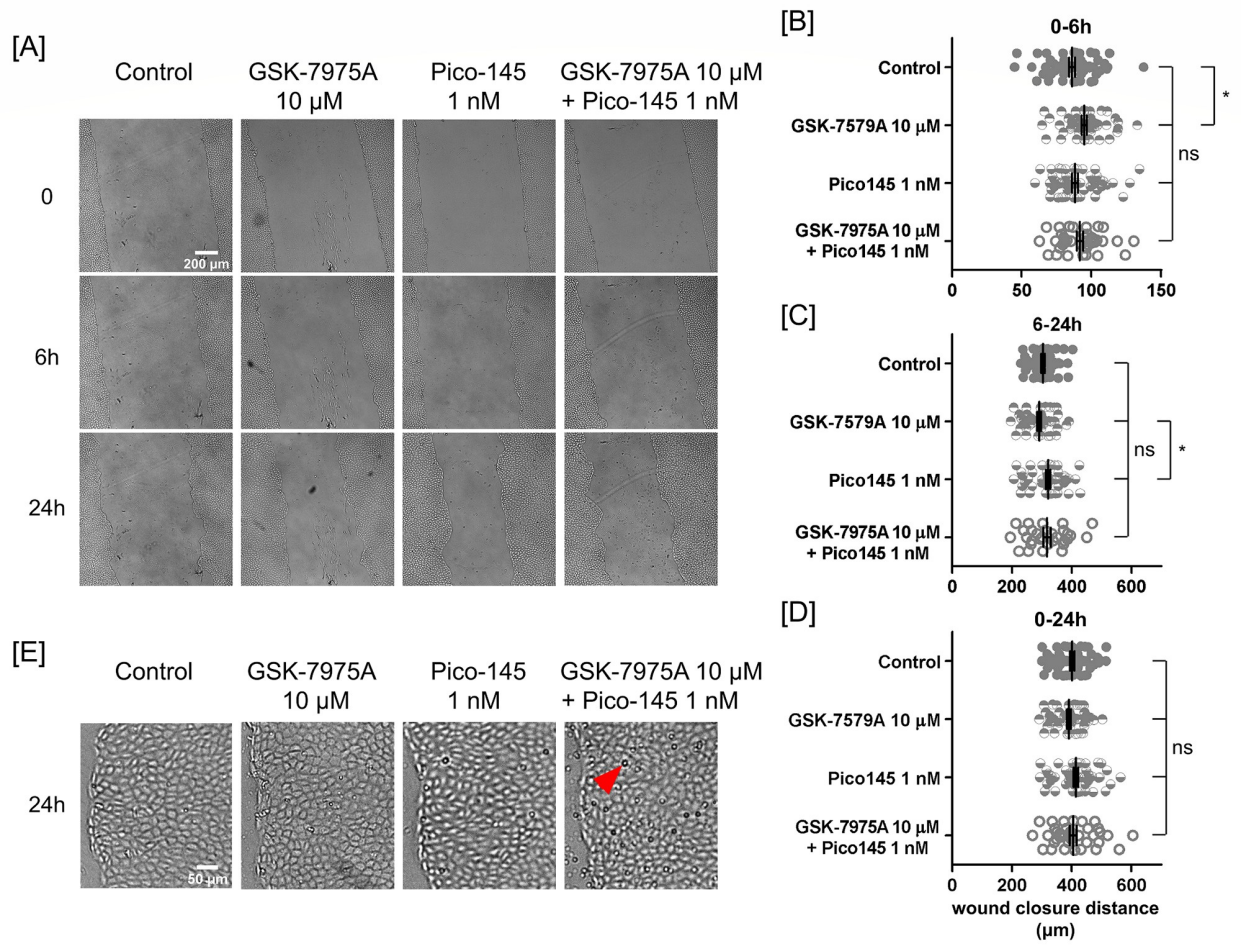
Inhibition of Orai1 and TRPC1/4 did not affect overall wound closure. **(A)** Brightfield images of scratched PAEC monolayers at 0, 6, and 24-hours in vehicle control, 10 μM GSK-7975A, 1 nM Pico145, and combined 10 μM GSK-7975A and 1 nM Pico145 conditions. Combined GSK-7975A and Pico145 treatment caused an accumulation of floating particles over 24 hours. Studies were all conducted in Na^+^-containing migration media. Scatter plots of wound closure distance in **(B)** 0–6, **(C)** 6–24, and **(D)** 0–24 hours. Data were reported as mean ± SEM. **(E)** Magnified view of the marginal regions of the control, GSK-7975A-treated, Pico145-treated, and both GSK-7975A- and Pico145-treated cells after 24 hours of wound closure. An arrowhead points to a floating particle. Statistical significance was assessed using one-way ANOVA with *Bonferroni post hoc* test (ns—not significant, *—P < 0.05).

Our previous study revealed that silence of Orai1 constitutively increases Na^+^ permeation through the store-operated Ca^2+^ entry channel [26]. To study the effect of inhibiting Orai1 activity on PAEC monolayer wound closure, we inhibited Orai1 with 10 μM GSK-7975A in the presence of extracellular Na^+^ [34]. Note that GSK-7975A also blocks smaller Ca^2+^ inward currents via Orai2 and Orai3 channels, respectively [41]. GSK-7975A treatment slightly enhanced scratch wound closure in the first 6 hours. However, there was no effect on wound closure after 6 hours or on overall wound closure (Fig 5A–5E).

To study the effect of Na^+^ permeation through the TRPC1/4 channel on PAEC monolayer wound closure, we inhibited TRPC1/4 with 1 nM Pico145 in the presence of extracellular Na^+^ [35]. Note that Pico145 also blocks TRPC4, TRPC5, and TRPC1/5 channels [35]. The results revealed that Pico145 treatment did not affect overall wound closure significantly (Fig 5A–5D). However, between 6 and 24 hours post scratch, Pico145 treatment caused slightly faster wound closure than did the inhibition of Orai1 (Fig 5C).

### Inhibition of both Orai1 and TRPC1/4 channels increases floating particles in the media

Over 24 hours, floating particles were hardly observed in the control, GSK-7975A-treated, and Pico145-treated monolayers (Fig 5A and 5E). By contrast, there were appreciable amounts of floating particles in the combined treatment of GSK-7975A and Pico145 (Fig 5A and 5E).

### Orai1 silencing transiently slows endothelial wound closure

**Fig 6:**
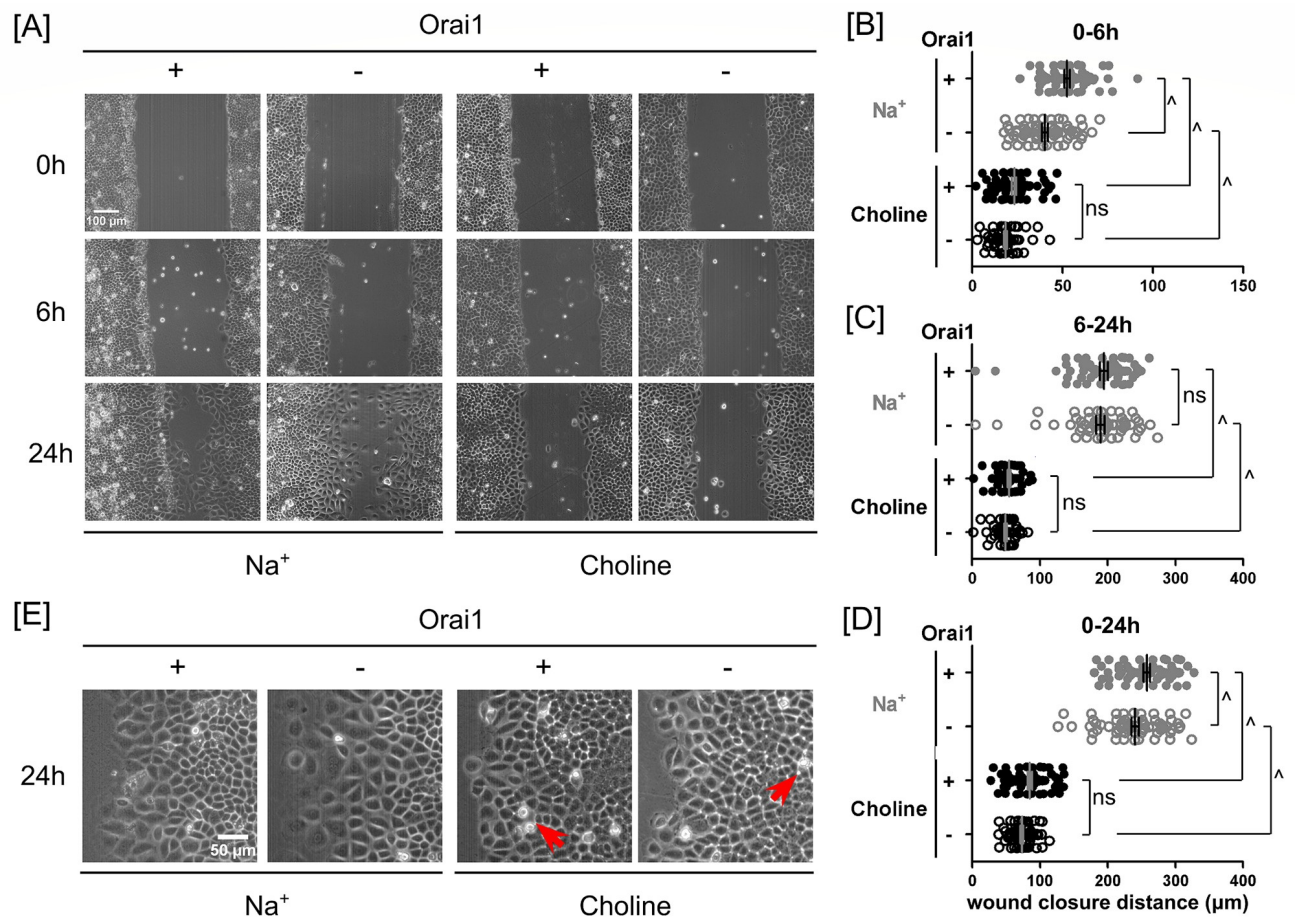
In the presence of extracellular Na^+^, Orai1 silencing slowed endothelial wound closure. **(A)** Phase-contrast images of scratched Orai1-expressing and -silenced PA2879 endothelial monolayers at 0, 6 and 24 hours in the presence and absence of extracellular Na^+^. Scatter plots of wound closure distance in **(B)** 0–6 hours, **(C)** 6–24 hours, and **(D)** 0–24 hours. In the presence of extracellular Na^+^, wound closure distances in Orai1-silenced PA2979 monolayers were less than the distances in Orai1-expressing monolayers. However, in the absence of extracellular Na^+^, the contribution of Orai1 was not significant. Data were reported as mean ± SEM. **(E)** Magnified view of the marginal regions of the Orai1-expressing and -silenced cells at 24 hours of wound closure in Na^+^- and choline-containing media. Cells near the wound edge spread larger in Na^+^- than in choline-containing media. Intercellular gaps were observed in the absence of extracellular Na^+^. Arrows point to typical interendothelial gaps. Statistical significance was assessed using one-way ANOVA with *Tukey’s post hoc* test (ns—not significant, ^—*P* < 0.0001).

To examine the effect of Na^+^ permeation through the Orai1-associated store-operated Ca^2+^ entry channel on PAEC monolayer wound closure, we genetically modified PA2879 cells to conditionally suppress Orai1 expression [25, 26]. In the presence of extracellular Na^+^, Orai1 silencing increased Na^+^ permeation through the store-operated Ca^2+^ entry channel [25, 26]. In the migration studies, we found that Orai1 silencing reduced about 25% wound closure in the first 6 hours but had no effect on wound closure between 6 and 24 hours (Fig 6B and 6C). In the absence of extracellular Na^+^, Orai1 silencing did not affect wound closure (Fig 6B–6D).

### Extracellular Na^+^, scratch-associated cellular injury, and Orai1 together facilitate the resealing of interendothelial gaps

**Fig 7:**
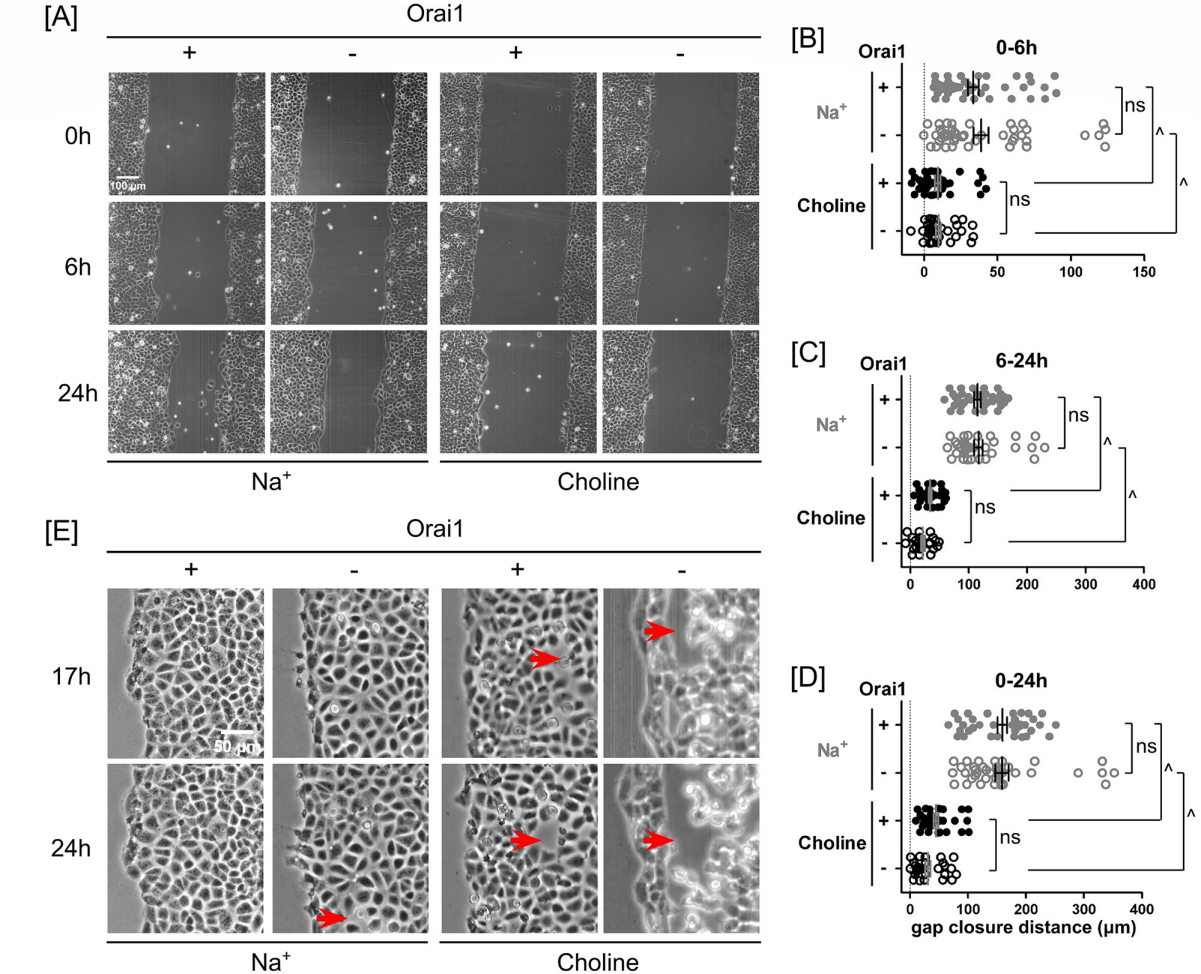
Extracellular Na^+^ promoted PAEC monolayer unscratched gap closure. The presence of both Orai1 and extracellular Na^+^ improved intercellular adhesion. **(A)** Phase-contrast images of Orai1-expressing and -silenced PA2879 monolayers at 0, 6 and 24 hours in the presence and absence of extracellular Na^+^. Scatter plots of gap closure distances in **(B)** 0–6 hours, **(C)** 6–24 hours, and **(D)** 0–24 hours. Replacing extracellular Na^+^ with choline significantly slowed PA2879 gap closure whereas silence of Orai1 had no effect on gap closure. **(E)** Magnified view of the marginal regions of Orai1-expressing and -silenced monolayers at 17 and 24 hours of migration in Na^+^- and choline-containing media. Without scratch-associated cellular injury, the marginal and submarginal cells were of similar size. Lack of extracellular Na^+^ and/or Orai1 compromised intercellular adhesion. Arrows point to typical interendothelial gaps. Statistical significance was assessed using one-way ANOVA with *Tukey’s post hoc* test (ns—not significant, ^—*P* < 0.0001).

In PA2879 monolayers, when migration was triggered by scratching, these interendothelial gaps transiently formed and resealed quickly in Na^+^-containing media. However, such gaps remained prominently visible in choline-containing media (Fig 6E). When monolayer migration was initiated without scratch-associated cellular injury, intercellular gaps were not visible in Orai1-expressing monolayers even up to 24 hours (Fig 7E). In contrast, larger interendothelial gaps were observed by 17 hours in Orai1-expressing cells incubated in choline media. This effect of choline was made worse by deleting Orai1. By 17 hours, these Orai1-silenced cells had already detached from the monolayer; the state of the monolayer further deteriorated in the last several hours of the experiment. These results indicates that extracellular Na^+^ is more important than Orai1 in preservation of intercellular adhesion.

### Extracellular Na^+^ is a dominant determinant of enhanced monolayer migration

To investigate the effect of Na^+^ permeation through the Orai1-associated store-operated Ca^2+^ entry channel on unscratched gap closure, we studied Orai1-expressing and -silenced PA2879 monolayers. We found that Orai1 silencing had no effect on gap closure over 24 hours (Fig 7A–7D). Comparing the monolayer migration between scratched and unscratched conditions in Na^+^-containing media, we found that gap closure distance reduced by 27% in unscratched condition. Comparing the monolayer migration in scratched condition between Na^+^ and choline media, we found that gap closure distance reduced by 52% in choline media. These two reductions revealed that the enhanced migration promoted by extracellular Na^+^ was stronger than that caused by scratch-associated cell injury (S1A and S1C Fig [https://doi.org/10.6084/m9.figshare.13818989https://sites.google.com/a/southalabama.edu/imbl/sodium_permeability_ning]).

### Scratch-associated cellular injury enhances monolayer migration

In Na^+^-containing media, scratch injury to Orai1-expressing PA2879 monolayers was associated with 36% increase in migration distance in the first 6 hours and 45% increase after 6 hours when compared to the unscratched counterparts (S1A and S1C Fig [https://doi.org/10.6084/m9.figshare.13818989https://sites.google.com/a/southalabama.edu/imbl/sodium_permeability_ning]). In contrast, similar scratch injury to Orai1-silenced PA2879 monolayers was associated with no effect on monolayer migration in the first 6 hours and 34% increase in migration distance after 6 hours (S1B and S1D Fig [https://doi.org/10.6084/m9.figshare.13818989]).

In choline-containing media, regardless of Orai1 expression, cellular injury contributed to faster monolayer migration (S1A–S1D Fig [https://doi.org/10.6084/m9.figshare.13818989]).

### In the presence of extracellular Na^+^, scratch injury is associated with enhanced spreading of marginal cells

When migration was triggered by a scratch in the presence of extracellular Na^+^, the marginal cells of both Orai1-expressing and -silenced PA2879 monolayers spread larger than did the cells located away from the wound edge (Figs 3A (left column) and 6E (left two columns)). However, when migration was triggered by removing a culture insert, the marginal and submarginal cells appeared to be of similar size (Fig 7E (left two columns)).

### Scratch evokes acute cytosolic Ca^2+^ and Na^+^ increases at the wound edge

In the spatial manner, scratch injury immediately increased both cytosolic Ca^2+^ and Na^+^ fluorescence intensity in control cells at the wound edge (S3A and S3B Fig [https://doi.org/10.6084/m9.figshare.13818989]). Cytosolic Ca^2+^ and Na^+^ quickly decreased in control cells within 50 μm from the wound edge. However, further deep into the control monolayer, only cytosolic Ca^2+^ in cells between 50 and 200 μm from the wound edge increased to form a second peak (S3A and S3C Fig [https://doi.org/10.6084/m9.figshare.13818989]). Cytosolic Na^+^ in control cells at the same region did not form a distinct submarginal peak (S3B and S3D Fig [https://doi.org/10.6084/m9.figshare.13818989]).

In the temporal manner, the surge of cytosolic Ca^2+^ and Na^+^ in control cells at the wound edge receded in 30 minutes (S3C and S3D Fig [https://doi.org/10.6084/m9.figshare.13818989]). The surged cytoplasmic Ca^2+^ and Na^+^ in control cells within 50 μm from the wound edge receded faster than those in control cells beyond 50 μm from the wound edge. The second Ca^2+^ peak was much short-lived and receded within 6 minutes (S3E Fig [https://doi.org/10.6084/m9.figshare.13818989]). In control cells beyond 200 μm from the wound edge, both cytosolic Ca^2+^ and Na^+^ elevated again after 30 minutes (S3A and S3B Fig [https://doi.org/10.6084/m9.figshare.13818989]).

### Orai1 activity contributes to scratch-evoked Ca^2+^ peak near the wound edge

To investigate the contribution of Orai1 activity to scratch-triggered changes in cytosolic Ca^2+^ and Na^+^, we added 10 μM GSK-7975A to PAEC monolayer to inhibit Orai1 [34]. Note that GSK-7975A also blocks smaller Ca^2+^ entry via Orai2 and Orai3 channels [41]. GSK-7975A treatment neither abolished the onset nor the decay of scratch-triggered Ca^2+^ peak at the wound edge, suggesting that Orai1 did not contribute to the Ca^2+^ influx or decay in that area (S3A, S3C and S3F Fig [https://doi.org/10.6084/m9.figshare.13818989]). However, GSK-7975A fully eliminated the second Ca^2+^ peak, suggesting that Orai1 in submarginal cells somehow responded to scratching leading to transient increases in cytosolic Ca^2+^. In the remaining monolayer, GSK-7975A treatment appeared to attenuate changes in cytosolic Ca^2+^.

GSK-7975A treatment did not fully abolish the Na^+^ peak or decay at the wound edge either (S3B and S3D Fig [https://doi.org/10.6084/m9.figshare.13818989]). In the remaining monolayer, GSK-7975A treatment also appeared to attenuate changes in cytosolic Na^+^.

## Discussion

Scratching endothelial monolayer relieves contact inhibition and triggers surviving cells near the wound edges to migrate and proliferate to heal the wound. The aim of this study was to identify the physiological impacts of Na^+^ permeation in PAEC scratch wound healing and unscratched collective migration. Here, we report six key findings from this study. 1) Extracellular Na^+^ is a critical determinant of endothelial cell migration in both scratch wound closure and unscratched collective migration models. 2) The processes of scratch-triggered wound closure contain two phases: a fast but rapidly slowing phase within 5 hours post-scratch, followed by a slow and stable phase from 5 to 24 hours. 3) Extracellular Na^+^ is necessary for lamellipodia formation and stabilization of intercellular adhesion during collective migration. 4) Na^+^ permeation via ENaC or both ENaC and NCX promotes endothelial wound closure. 5) Ca^2+^ and Na^+^ permeation via Orai1, TRPC1/4, or both Orai1 and TRPC1/4 did not have a significant long-term effect on wound closure. 6) In the presence of extracellular Na^+^, Orai1 expression, but not acute Orai1 activation, promoted scratch-triggered short-term wound closure.

Traditional *in vitro* assays to study collective migration of cellular monolayer involve scratching a confluent monolayer and then observing its ability to heal, *i*.*e*., recover the wounded area. In case of an endothelial monolayer, the surviving cells near the wound edge lead the process of wound closure by protruding lamellipodia and triggering proliferation and migration of cells to cover the wounded area [42]. In our migration study, we found that Na^+^ ions in the migration media were critical to protrude lamellipodia and to sustain wound closure. The dynamics of PAEC monolayer scratch wound closure contained two distinct phases (Fig 2G, 2I and 2J). We used term “first phase” to describe migration in 0–6 hours and use term “second phase” to describe migration in 6–24 hours (Figs 4–7 and S1 Fig).

Cells tightly regulate the spatial and temporal spread of cytosolic Na^+^ and Ca^2+^ signals [7, 14, 15, 20, 22, 23]. Hence, to study the contribution of Na^+^ permeation to PAEC monolayer wound closure and collective migration, we blocked extracellular Na^+^ influx by replacing Na^+^ ions with choline in migration media. Such substitution has been found to hyperpolarize the membrane potential of non-migrating cells in a confluent corneal endothelial monolayer and to enhance the amount of F-actin along the intercellular borders [43]. In a wounded monolayer, such hyperpolarization reduces both the amount of actin cables and the extent of lamellar activity in cells near the wound edge [22]. We observed similar suppression of lamellipodia in PAECs at the wound edge in choline-containing media. PAEC monolayers also migrated much slower in choline- than in Na^+^-containing media. After 12 hours of wound closure, these cells eventually became near stagnant even in the presence of extracellular Ca^2+^ (Fig 2G). These observations emphasize the necessity of extracellular Na^+^ in the maintenance of cellular migration machinery and cellular polarity.

Lamellipodial activity similar to that observed at the migration front is also responsible for sealing intercellular gaps [44]. As such, the lack of extracellular Na^+^ is expected to affect not only PAEC monolayer migration but also the ability to sustain and reinstate intercellular adhesion. We observed transient interendothelial gaps around dividing cells immediately prior to cell division. In the presence of extracellular Na^+^, these gaps resealed promptly. However, in the absence of extracellular Na^+^, some adjacent interendothelial gaps coalesced to form sustained larger gaps (Fig 3). Such involvement of extracellular Na^+^ in the intercellular adhesion of PAECs is a novel finding and further studies are needed to map the associated molecular mechanisms.

In cells close to the wound edge, scratch injury triggers an increase in ENaC expression which facilitates Na^+^ influx thereby depolarizing the membrane potential [15, 22]. Even 24 hours later the membrane potential of surviving wound-edge cells remains depolarized, and cytosolic Na^+^ remains elevated [22]. Coupling between ENaC and NCX alters cytosolic Na^+^ and Ca^2+^ concentrations and causes slow Ca^2+^ wave [15, 23]. The impact of inhibiting ENaC by amiloride or both ENaC and NCX by benzamil on PAEC wound closure was significant; wound closure reduced by more than 30% (Fig 4D). Remarkably, the combined effect of inhibiting both ENaC and NCX seemed to occur in the late phase (6–24 h) of wound closure (Fig 4B–4D) where it appeared to trigger an accumulation of floating particles in the media (Fig 4E). The diameter of these particles was about half of the diameter of cells, suggesting that these particles may be the unattached products after cell division.

Orai1 is a subunit of a highly Ca^2+^-selective channel that is activated upon endoplasmic reticulum Ca^2+^ depletion [45–48]. In the absence of Orai1, the store-operated Ca^2+^ entry channel becomes less Ca^2+^ selective by increasing Na^+^ intake without reducing Ca^2+^ entry in confluent and non-migrating PAEC monolayers [25, 26]. In human umbilical vein endothelial cells, Orai1 silencing causes more than 50% reduction in the number of cells migrated through the Boyden chamber [19]. In individual human embryonic kidney (HEK293) cells, Orai1 silencing leads to fewer focal adhesions per cell, and these focal adhesions are larger and closer to the cell border [20]. In sparse cell populations, the Orai1 influence on focal adhesions causes more than 30% reduction in spreading area and about 20% reduction in migration speed [20].

Previously, our group found that Orai1 constitutively interacts with TRPC4 and interacts with both TRPC1 and TRPC4 following thapsigargin-induced activation of the store operated Ca^2+^ channel [25]. Activation of the channel increases channel Ca^2+^ selectivity and causes interendothelial gap formation [25]. To examine the contribution of Orai1 and TRPC1/4 activity to PAEC monolayer wound closure, we inhibited Orai1 and TRPC1/4 using 10 μM GSK-7975A and 1 nM Pico145, respectively. Note that GSK-7975A also blocks smaller Ca^2+^ entry via Orai2 and Orai3 channels, whereas Pico145 blocks TRPC4, TRPC5, and TRPC1/5 channels as well [35, 41]. The results showed that GSK-7975A, Pico145, or combined GSK-7975A and Pico145 treatments did not affect PAEC monolayer wound closure (Fig 5B–5D). Nevertheless, the combined treatment caused an accumulation of floating particles in the media (Fig 5E).

We examined the contribution of Orai1 to wound closure using two approaches: one is gene silencing, the other is pharmacological inhibition. Using the genetic approach, we also investigated unscratched gap closure. Such genetic and pharmacological approaches have inherent differences. The genetic approach involved the inducing expression of Orai1 shRNAs to block Orai1 gene expression for 72 hours before generating a gap within a confluent PAEC monolayer. During collective migration, shRNA expression was stopped, and thus Orai1 expression was resumed. We did not monitor the changes of other channel subunits during Orai1 silencing for 72 hours, nor did we monitor the extent of Orai1 reexpression during migration for 24 hours. In contrast, the pharmacological approach started with addition of 10 μM GSK-7975A immediately before scratching or 15 minutes prior to the scratch injury to inhibit Orai1 activity and maintained GSK-7975A in the media during wound closure. It is important to note that GSK-7975A also inhibits Orai2 and Orai3 channels, respectively [41]. These two approaches produced different short-term closure responses in the first phase: while Orai1 silencing caused 25% reduction in wound closure in the presence of extracellular Na^+^, GSK-7975A inhibition caused 10% enhancement in wound closure (Figs 5B and 6B). Nevertheless, in the second phase, both approaches did not affect wound closure when compared to their corresponding controls (Figs 5C and 6C). Overall, both approaches had rather small effects on wound closure in 24 hours (Figs 5D and 6D). Due to the lack of extracellular Na^+^, PAEC monolayers migrated much slower during both scratch wound closure and unscratched gap closure when compared to their Na^+^ counterparts. Overall, the contribution of Orai1 expression to PAEC monolayer wound closure is short-term and closely associated with cellular injury and extracellular Na^+^ (Figs 6 and 7).

The contribution of Orai1 to cellular migration comes from clustering of Orai1 at the cell border. Such clustering activates Ca^2+^ influx that converts extracellular cues into cytosolic signals which steer directional cell migration [14, 20]. As mentioned earlier, in a confluent and non-migrating PAEC monolayer, Orai1 silencing increases baseline Na^+^ leak into cells [26]. To examine how Orai1 regulates Ca^2+^ and Na^+^ permeation in response to scratching, we monitored cytosolic Ca^2+^ and Na^+^ transients immediately after scratching for 30 minutes in the presence and absence of Orai1 inhibitor GSK-7975A (S3A–S3F Fig [https://doi.org/10.6084/m9.figshare.13818989]). In control samples, cellular injury triggered an immediate increase and then a gradual decrease in cytosolic Ca^2+^ in marginal cells. Simultaneously, cytosolic Na^+^ in marginal cells also experienced a prompt increase and then a gradual decrease. GSK-7975A inhibition caused small effects on Ca^2+^ and Na^+^ increases and decay in marginal cells. In control cells between 50 and 200 μm from the wound edge, cytosolic Ca^2+^ elevated to form a distinct Ca^2+^ peak in immediate response to scratching. This Ca^2+^ peak was transient and quickly decayed to baseline in about 6 minutes. GSK-7975A inhibition fully abolished this Ca^2+^ peak. However, there was no such scratch-induced Na^+^ peak in this submarginal region. Taken together, Orai1-associated increases in cytosolic Ca^2+^ and Na^+^ transients due to scratching were consistent with the observed small short-term effect of Orai1 activity on PAEC monolayer wound closure (Fig 5B).

In epithelial monolayers, scratch-associated cellular injury enhances monolayer migration speed [49]. In PAEC monolayers, we observed that scratch-associated cellular injury promoted wound closure (S1A–S1D Fig [https://doi.org/10.6084/m9.figshare.13818989]). However, the contribution of extracellular Na^+^ was greater than that of a scratch to promote wound healing. Interestingly, scratching still promoted wound closure faster than unscratched gap closure in the absence of extracellular Na^+^. Unscratched PAEC monolayers closed gaps faster in Na^+^- than in choline-containing media. In the presence of extracellular Na^+^, Orai1 expression only enhanced PAEC scratch wound closure in a short term, and it did not contribute to unscratched gap closure (Figs 6B and 7B). In the absence of extracellular Na^+^, Orai1 expression contributed to neither scratch wound closure nor unscratched gap closure (Figs 6B–6D and 7B–7D).

In summary, permeation of extracellular Na^+^ is necessary for efficient and cohesive migration of PAEC monolayers. Such necessity appeared to emerge from the influence of Na^+^ on lamellipodial activity and intercellular cohesion. Among the examined Na^+^ channels, Na^+^ permeation through ENaC produced prompt and largest effect, Na^+^ permeation through NCX produced small and delayed effect, Na^+^ permeation through Orai1 produced small and short-term effect, and Na^+^ permeation through TRPC1/4 produced insignificant effect. Note that GSK-7975A also inhibits Orai2 and Orai3 channels, whereas Pico145 also blocks TRPC4, TRPC5, and TRPC1/5 channels. Over 24 hours, blocking multiple Na^+^ transport channels triggered accumulation of floating particles in the media. These observations emphasize the need to examine Na^+^ permeation-associated changes in intercellular mechanical signals, intercellular tractions, and cellular moments [39, 50]. The data also brings a spotlight on the need to identify the potential downstream Na^+^ effectors that respond to endothelial injury and contribute to wound repair.

## Supporting information

S1 Fig Comparison of PAEC monolayer migration triggered with and without scratching. Scatter plots of migration distances of PA2879 monolayers triggered by scratching (S) or without scratching (NS) in the presence and absence of extracellular Na^+^ in (A) Orai1-expressing and (B) -silenced conditions over 0–6 hours. Panels (C) and (D) show the scatter plots of the corresponding closure distances over 6–24 hours. Statistical significance was assessed using one-way ANOVA with *Tukey’s post hoc* test (ns—not significant, ^—*P* < 0.0001). [https://doi.org/10.6084/m9.figshare.13818989].
(TIF)

S2 Fig Distance transform-based data analysis of cytosolic Ca^2+^ and Na^+^ intensity as a function of distance from wound edge. The image of cellular monolayer was segmented using local variance intensity. This allowed creation of a binary image that distinguished monolayer from the cell-free area. A distance transform of this binary image was used to obtain contours spaced ~30 μm apart starting with the migration front being the first contour. The area contained between two successive contours was defined as the band across which the Ca^2+^ and Na^+^ intensities were collected to compute the mean and SEM of the intensities in that band. [https://doi.org/10.6084/m9.figshare.13818989].
(TIF)

S3 Fig Spatiotemporal transients of cytosolic Ca^2+^ and Na^+^ in responding to scratching in the presence and absence of Orai1 inhibition. Mean cytosolic **(A)** Ca^2+^ and **(B)** Na^+^ transients within each ~30 μm band (see S2 Fig [https://doi.org/10.6084/m9.figshare.13818989]) in vehicle control and 10 μM GSK-7975A conditions at t0 (right after scratching) and t30 (30 minutes after scratching). Percentage changes in the cytosolic **(C)** Ca^2+^ and **(D)** Na^+^ transients within 30 minutes post scratch. In **(C)**, the dashed line indicates mean + SEM. Statistical significance was assessed using linear regression. Cytosolic Ca^2+^ decayed in **(E)** control and **(F)** GSK-7975A conditions within 30 minutes post scratch. In control monolayer **(E)**, data at t = 2, 3, 4, 6, 10, and 30 minutes were significantly different from the data at t = 0. Data at t = 4, 6, and 10 minutes were significantly different from the data at t = 1 minute. In GSK-7975A-treated monolayer **(F)**, there was no significant difference between data at any two-time points. Each curve was normalized based on the corresponding baseline intensity before the scratch injury. Statistical significance was assessed using one-way ANOVA with *Bonferroni post hoc* test. (ns—not significant, *—*P* < 0.05). [https://doi.org/10.6084/m9.figshare.13818989].
(TIF)

S1 Movie Scratch wound closure in Na^+^-containing DMEM within 24 hours. PAEC confluent monolayer was scratched and kept in 2% regular DMEM for wound closure assay. The cell dish was incubated in a humidified chamber at 37℃ supplied with 5% CO_2_ on a microscope stage. The wound closure procedure was recorded at 10-minute intervals using an Olympus IX70 Phase Contrast Inverted Microscope with an Olympus LCPlanFl 20× objective. [https://doi.org/10.6084/m9.figshare.13818989].
(AVI)

S2 Movie Scratch wound closure in choline-containing DMEM within 24 hours. PAEC confluent monolayer was scratched and maintained in 2% choline-containing DMEM for wound closure. Na^**+**^ was substituted by choline in the media. The rest settings were the same as those in the Na^+^ condition. [https://doi.org/10.6084/m9.figshare.13818989].
(AVI)
