# Supplementary figures and images for: Impact of Na+ permeation on collective migration of pulmonary arterial endothelial cells
Source: PLoS One. 2021 Apr 23;16(4):e0250095. doi: 10.1371/journal.pone.0250095 (PMC8064576; doi:10.1371/journal.pone.0250095)

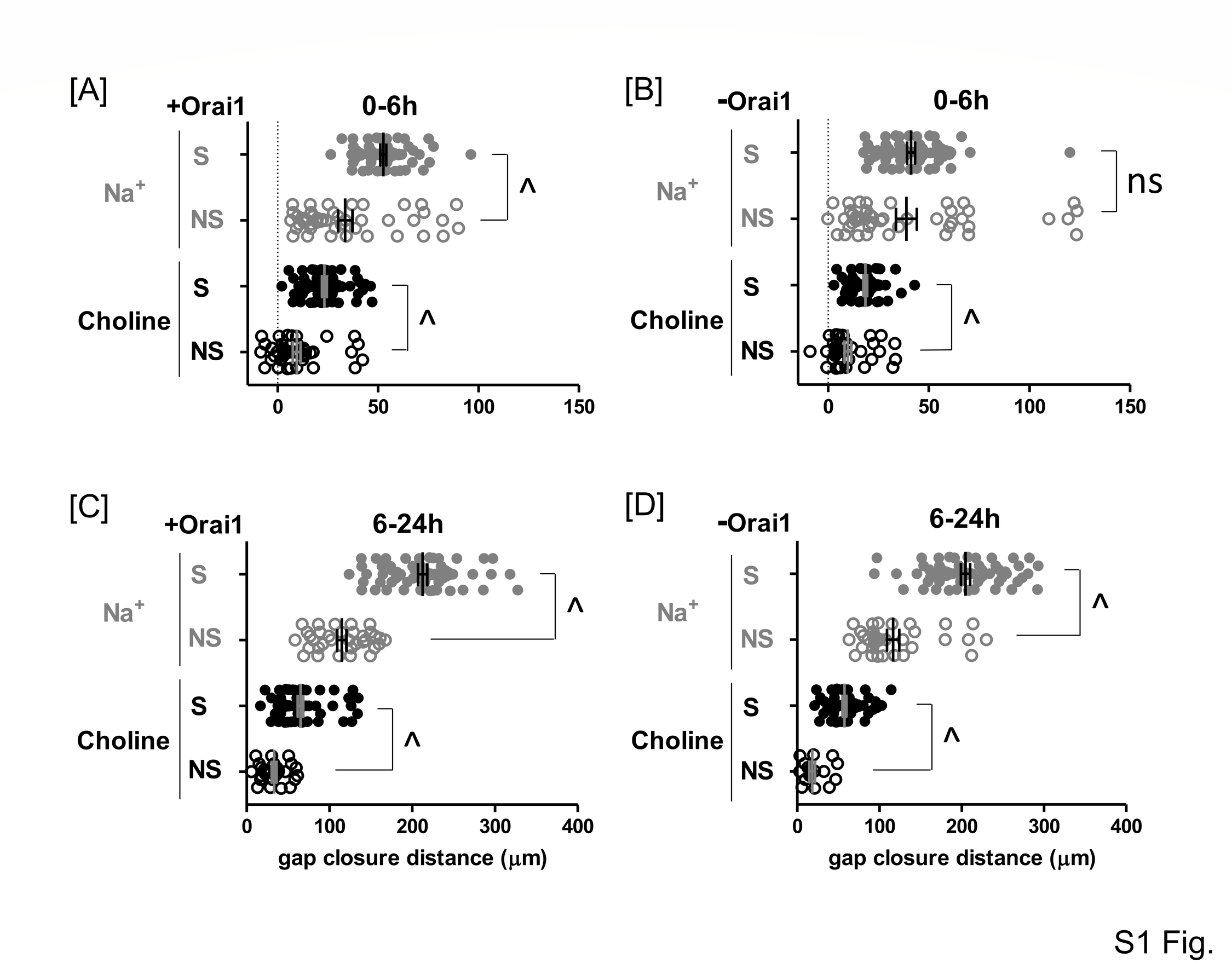

Supplement: S1 Fig — Scatter plots of migration distances of PA2879 monolayers triggered by scratching (S) or without scratching (NS) in the presence and absence of extracellular Na+ in (A) Orai1-expressing and (B) -silenced conditions over 0–6 hours. Panels (C) and (D) show the scatter plots of the corresponding closure distances over 6–24 hours. Statistical significance was assessed using one-way ANOVA with Tukey’s post hoc test (ns—not significant, ^—P < 0.0001). [https://doi.org/10.6084/m9.figshare.13818989]. (TIF) [file pone.0250095.s001.tif]

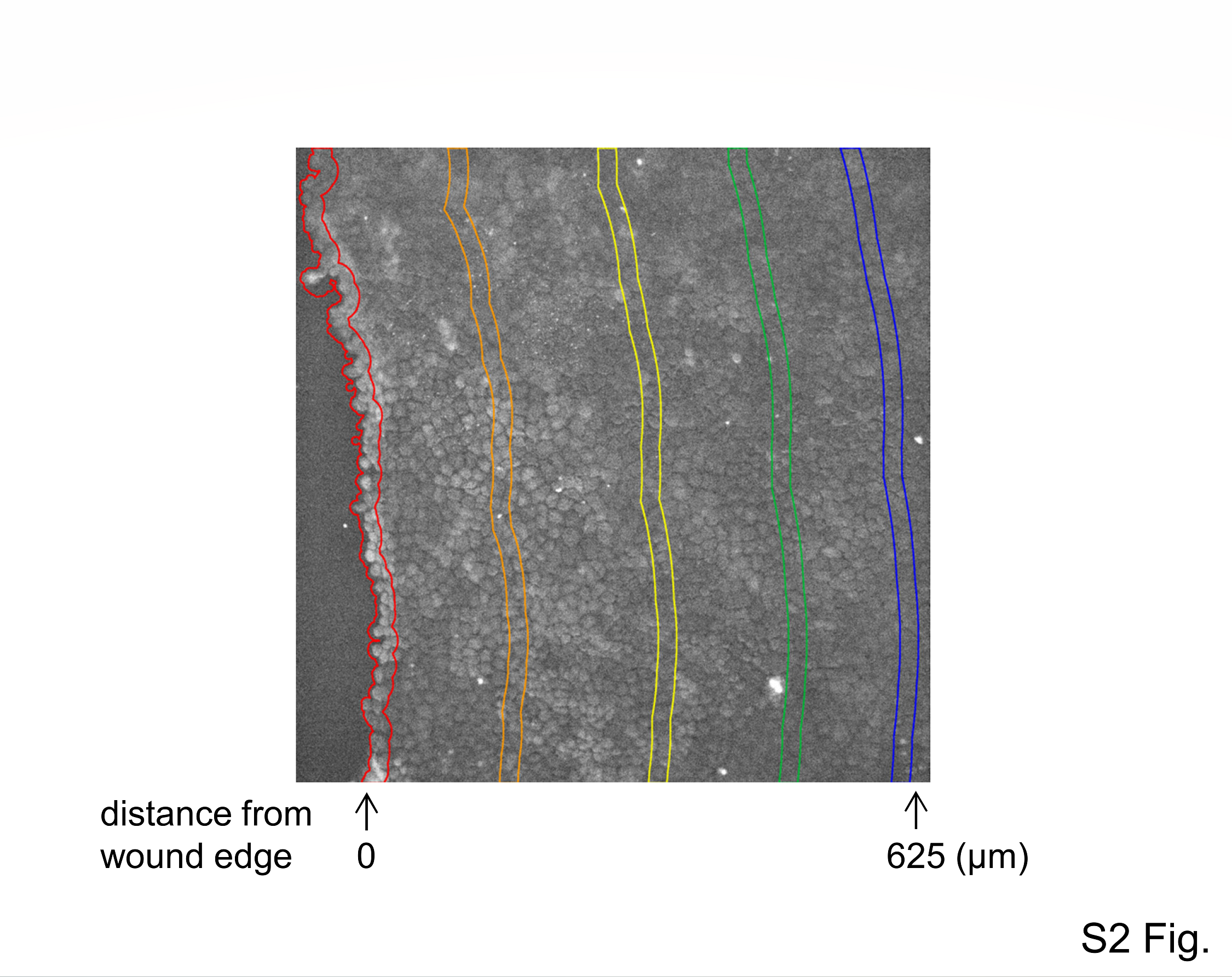

Supplement: S2 Fig — The image of cellular monolayer was segmented using local variance intensity. This allowed creation of a binary image that distinguished monolayer from the cell-free area. A distance transform of this binary image was used to obtain contours spaced ~30 μm apart starting with the migration front being the first contour. The area contained between two successive contours was defined as the band across which the Ca2+ and Na+ intensities were collected to compute the mean and SEM of the intensities in that band. [https://doi.org/10.6084/m9.figshare.13818989]. (TIF) [file pone.0250095.s002.tif]

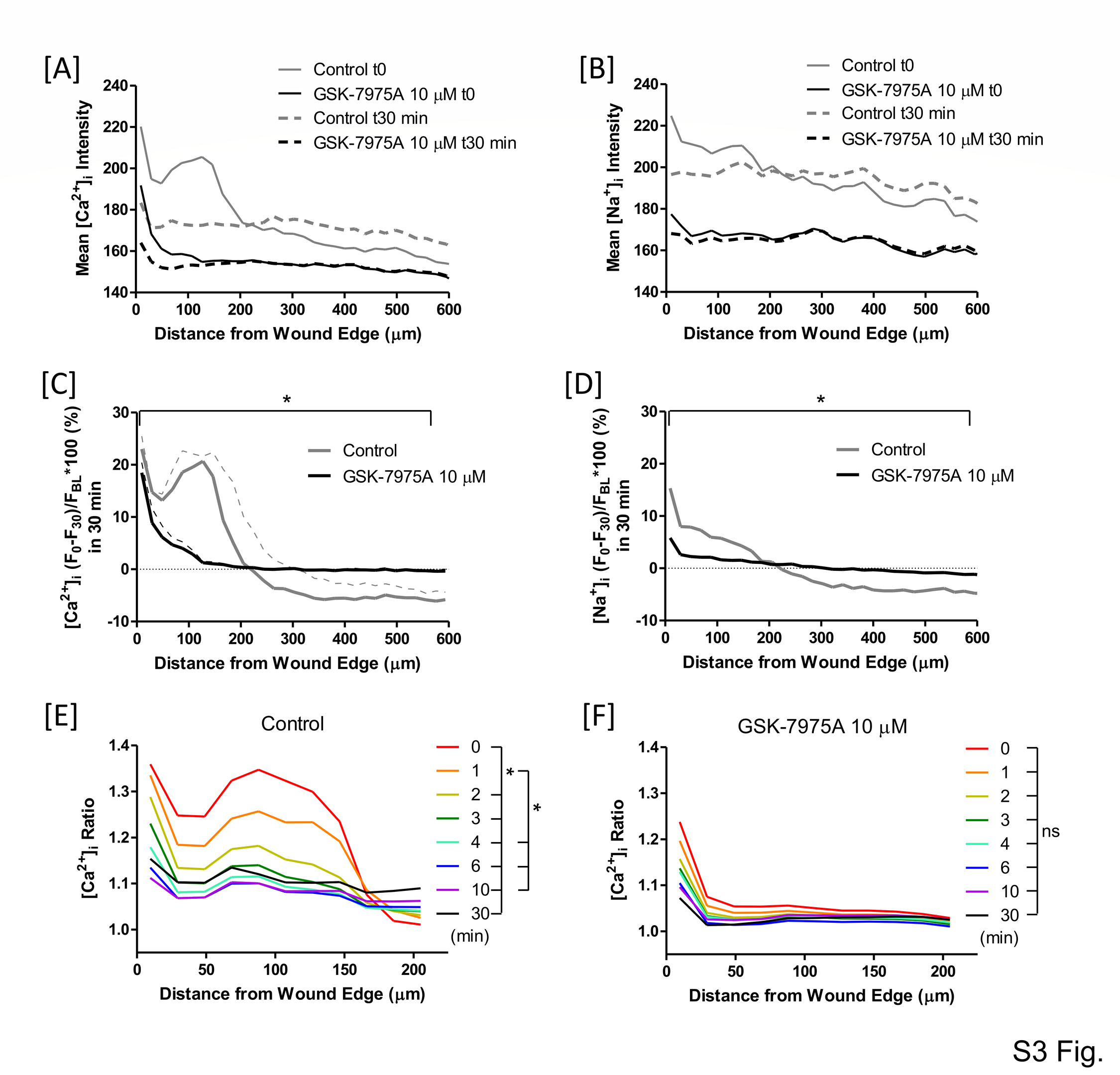

Supplement: S3 Fig — Mean cytosolic (A) Ca2+ and (B) Na+ transients within each ~30 μm band (see S2 Fig [https://doi.org/10.6084/m9.figshare.13818989]) in vehicle control and 10 μM GSK-7975A conditions at t0 (right after scratching) and t30 (30 minutes after scratching). Percentage changes in the cytosolic (C) Ca2+ and (D) Na+ transients within 30 minutes post scratch. In (C), the dashed line indicates mean + SEM. Statistical significance was assessed using linear regression. Cytosolic Ca2+ decayed in (E) control and (F) GSK-7975A conditions within 30 minutes post scratch. In control monolayer (E), data at t = 2, 3, 4, 6, 10, and 30 minutes were significantly different from the data at t = 0. Data at t = 4, 6, and 10 minutes were significantly different from the data at t = 1 minute. In GSK-7975A-treated monolayer (F), there was no significant difference between data at any two-time points. Each curve was normalized based on the corresponding baseline intensity before the scratch injury. Statistical significance was assessed using one-way ANOVA with Bonferroni post hoc test. (ns—not significant, *—P < 0.05). [https://doi.org/10.6084/m9.figshare.13818989]. (TIF) [file pone.0250095.s003.tif]
